# Assessment of Health Needs in Children with Congenital Upper Limb Differences in Nicaragua: Community Case Study

**DOI:** 10.3389/fpubh.2017.00123

**Published:** 2017-05-24

**Authors:** Maria F. Canizares, Jairo J. Rios Roque, Gabriel Ramos Zelaya, Michelle A. James

**Affiliations:** ^1^School of Public Health and Health Sciences, University of Massachusetts Amherst, Amherst, MA, USA; ^2^Manuel de Jesus Rivera “La Mascota” Hospital, Department of Orthopedic Surgery, Managua, Nicaragua; ^3^Department of Orthopedic Surgery, Shriners Hospital for Children Northern California, Sacramento, CA, USA

**Keywords:** global health, partnership, community, case study, needs assessment, congenital differences, upper limb, Nicaragua

## Abstract

Congenital anomalies are prevalent in Nicaragua, and disability is estimated to be 10% in the general population. We studied children with congenital upper limb differences, as they are vulnerable to disability. This case study documents a collaborative effort between American and Nicaraguan orthopedic surgeons to determine unmet health needs of children with congenital upper limb differences at Hospital Manuel de Jesus Rivera (“La Mascota” Hospital) in Nicaragua, with the goal of developing programs that successfully address these needs within the context of the priorities of the community. Participants were recruited during one of the biannual pediatric hand specialty clinics held by a partnership of pediatric hand surgeons and occupational therapists under the auspices of Health Volunteers Overseas (La Brigada de las Manos, or “La Brigada”) and Nicaraguan orthopedic surgeons. Structured interviews were performed with 34 parents or caregivers of patients with the diagnosis of a congenital upper limb difference. Parents were asked to rank the social, economic, environmental, and biological factors that determine health according to priority. Using the Hanlon Method for prioritizing health problems, in consultation with local providers and the program director of La Brigada, five needs were identified: (1) improvements in access to specialized care from hand surgeons and (2) rehabilitation specialists; (3) improvements in upper extremity function; (4) access to transportation; and (5) improvement in physical activity and sports participation. Based on the results of this needs assessment, we learned that some of the needs were already part of the ongoing work of the partnership, but in addition, more needs became evident; for that reason, local health care providers and members of La Brigada identified potential solutions to these needs and are currently working to translate these in future interventions.

## Introduction

Partnerships between local health-care professionals with international allies can improve health in developing countries, especially when they are based on equal participation, knowledge contribution, and focus on important community health issues (1). The challenge remains that collaborative alliances do not have clear roles and responsibilities when starting new programs. To that end, in order to establish and sustain a successful partnership with long-lasting results, developing a clear and common purpose among all stakeholders, including the community, is paramount when designing new interventions. One of the goals of global health partnerships is to prevent disability (2), especially in high-risk populations such as children. The purpose of this case study is to document a collaborative effort between American and Nicaraguan orthopedic surgeons to determine the unmet health needs of children with congenital upper limb differences, with the goal of developing a plan of action that successfully addresses these needs within the context of the priorities of the community.

## Background and Rationale

According to the World Health Organization (WHO), “Health is a state of complete physical, psychological, and social wellbeing and not simply the absence of disease or infirmity” (3). Based on this model, public health practitioners assess the needs of a population to identify biological, socioeconomic, and environmental risk factors and their relationship with disease. As health needs are identified, the next step is to plan comprehensive interventions to meet these needs and improve health as result.

Disability is a current public health concern in national and international agendas, which arises from the disturbance of anatomical structure or function and is highly influenced by social environment (4). As a result, people with disabilities require more health-care resources and may have more unmet needs than those without limitations (5). In developing countries, negative interactions between an individual with a health condition and their contextual environment aggravate the risk of disability. Children with congenital anomalies are particularly vulnerable to disability when their physical impairment is compounded by low socioeconomic status and lack of health-care resources.

According to WHO, congenital anomalies are prevalent in Nicaragua (6), and rates of disability of 10.2% are reported by the Pan American Health Organization (7). Unfortunately, relevant data about congenital musculoskeletal conditions are lacking. However, we know that children and adolescents with congenital upper limb differences experience variable degrees of physical impairment that can limit their ability to perform physical activity and disrupt of their emotional and social functioning (8). Depending on the level of involvement, a congenital difference can range from conditions that cause minimal impact on functioning to more complex conditions with potentially more disability (9). To begin identifying strategic priorities, the author was invited to participate as a volunteer to conduct a needs assessment of patients with congenital upper limb differences, as they are considered at high risk of disability. By recognizing their needs in the context of broader social, economic, and environmental determinants of health, this assessment will guide the development of future interventions.

## Collaborative Partnership

Under the auspices Health Volunteers Overseas (HVO), a non-profit organization dedicated to improving the availability and quality of health care through the education, training, and professional development of the health workforce in resource-scarce countries (10), a partnership developed in 2009 between an American team of hand surgeons and occupational therapists called “La Brigada de las Manos” with a team of Nicaraguan orthopedic surgeons from Fernando Velez Paiz Hospital. In 2014 after an earthquake destroyed this hospital, all personnel were transferred to Manuel Jesus Rivera “La Mascota” Hospital where La Brigada has continued to provide care on a biannual basis. Since the first Brigada de las Manos trip, the need to do more than just providing surgical care twice a year was evident, so the Brigada also undertook training of a local orthopedic surgeon to provide surgical treatment to simple upper extremity pathologies independently between Brigada trips and serve the as local hand surgeon. This training is still underway (11). Pediatric hand specialty clinics are still held twice a year with help of La Brigada de las Manos and the hand surgeon in training, in order to address more complex hand pathology.

## Setting

The needs assessment was conducted in Hospital Infantil Manuel de Jesus Rivera “La Mascota” located in Bo. Ariel Darce, Distrito V, Managua, Nicaragua. This pediatric hospital was established in 1982 in Nicaragua’s capital as a teaching governmental hospital that offers services free of charge and is administered by the Ministry of Health (Ministerio de Salud, or MINSA). The hospital not only offers services to the city but also receives referrals from the entire country making this institution the main referral center for children and adolescents less than 15 years of age. The Department of Orthopedic Surgery, where the need assessment took place, was recently transferred to “La Mascota” as above, and currently includes six staff surgeons, one of whom is dedicated to the treatment of upper limb conditions as above.

## Methods

This needs assessment evaluation employed structured interviews to obtain insight from families of children from 0 to 15 years of age with the diagnosis of a congenital upper limb difference, who sought care in La Mascota Hospital during the La Brigada visit in October 2016. Parents were questioned using a non-validated interview created for the purpose of this needs assessment. The interview was structured in five sections related to (1) demographic variables; (2) environmental factors such as water sanitation, housing, transportation, security, and access to health care services; (3) socioeconomic factors including education, employment, and income; (4) individual behavioral factors as nutrition, physical activity, and habits; and (5) biological factors such as the presence of a medical condition or history of genetic diseases. These sections were developed to have three possible responses: a positive outcome, a neutral outcome, and a negative outcome. For example, when asking about family monthly income, the possible responses included positive incomes (allowed savings), neutral (expenses equal income), or negative (expenses surpassed income). At the end of the interview, parents were asked to enumerate the most important needs for their family, and when more than one need was designated, they were asked to rank the need according to priority in the context of their life.

In addition to the interview, Spanish translations of Patient Reported Outcome Measurement Information System (PROMIS) questionnaire Pediatric Item Bank short form of upper extremity function were used for this assessment (12). Parent proxy-reported health measures were collected from patients older than 5 years of age and self-reports from children older than 8 years. PROMIS questionnaires were scored using the Assessment Center Scoring Manuals (13). Data collection was performed by a fluent Spanish speaking researcher to avoid language barriers. Informed consent was obtained from all participants prior to the interview. This study received approval from the University of Massachusetts IRB board and La Mascota Hospital authorities.

Demographic characteristics, determinants of health, and PROMIS scores were summarized for all patients and parents using descriptive statistics. The sections from the interview, related to both environmental and socioeconomic factors as well as individual behaviors and biological determinants of health were ranked according to the percentage of negative outcomes. If the average for PROMIS scores was less than 50 for peer upper extremity function domains, they were considered areas of need. A list of needs was created based on responses from interviews and questionnaires. In order to select the most important need, a Hanlon’s priority matrix method was used similar to previous program planning strategies (14). This prioritization technique requires rating each health problem identified on a scale from 0 to 10 on the following criteria: size of health problem (A), magnitude or seriousness of health problem (B), and effectiveness of potential interventions (C) (see Table 1). The size of the problem was assigned based of the percentage of negative outcomes obtained from the interviews; the seriousness of the problem was calculated taking into consideration the demand from parents when they were asked to rank the needs according to priority; and the effectiveness rating was assigned by consensus expert opinion of collaborators from La Brigada. A feasibility “PEARL” test was performed before calculating the total score; this test assigns a score of 1 if all the components of feasibility have a favorable response; however, if a response is negative, then a 0 is assigned. Finally, based on the three criteria rankings assigned to each health problem and the PEARL test, scores were calculated using the following formula: priority score = [A + (2 × B)] × C × PEARL.

**Table 1:** **Criteria rating of the Hanlon method**.

Rating	Size problem % of negative outcome	Magnitude % parental demand	Effectiveness of current La Brigada interventions (%)
9 or 10	>25	>25	80–100
7 or 8	10–24.9	10–24.9	60–80
5 or 6	1–9.99	1–9.99	40–60
3 or 4	0.1–0.99	0.1–0.99	20–40
2 o 1	0.01–0.09	0.01–0.09	5–20
0	<0.01	<0.01	<5

## Results

### Demographics

Thirty-four patients with a diagnosis of an upper limb difference were screened for this study, (19 males, 15 females). Patient median age was 30 months (IQR 18–63). The right side was most commonly affected, and unilateral conditions accounted for 68%. According to the Oberg–Manske–Tonkin classification for congenital differences, malformations in the hand were the most numerous group (41%) with radial polydactyly as the most common condition screened (15%). Parents of six patients did not complete the interview due to lack of time availability, and one parent did not want to provide socioeconomic information. Parents of 27 patients consented to participate and provided information centered about needs, including environmental, socioeconomic, behavioral, and biological factors determining health.

### Determinants of Health

#### Environmental Factors

Access to sources of water was limited; three (11%) families had to walk a considerable distance to obtain water from wells. While more families reported they had access to electricity, there was still one family (4%) without this service and instead used coal, biofuels, or candles to light their house. All respondents used public transportation, but seven (26%) of the parents reported having difficulties traveling to La Mascota Hospital. Finally, none of the families had insurance coverage, and all received medical attention in public institutions funded by the Nicaraguan government. The provision of basic health-care services was considered by the majority to be accessible and efficient; however, half of the respondents complained that prescribed drugs were only partially covered by the public health system. In addition, 22 (82%) parents reported difficulty accessing specialized care; in particular, this group was concerned that it was difficult to obtain access to a pediatric hand surgeon between La Brigada visits.

#### Socioeconomic Factors

A total of 19 (70%) of the respondents reported that the father economically supported the family; of those, 13 (68%) were full-time employees. Parents complained that monthly income varied under several circumstances, and nine (33%) respondents reported an unstable income. Regarding the highest level of education in the household, 19 (70%) of parents had completed primary education and started high school, and in one household, none of the parents (4%) had finished primary education.

#### Individual Behavioral Factors

According to five (19%) of the parents, nutrition in the family was not balanced and included a low proportion of fruit and vegetables. Similarly, 15 (56%) families mentioned that at least 1 member of the household had over or underweight. When asked about physical activity in children, seven (25%) of parents stated that their child performed moderate physical activity for less than 3 h per week.

#### Biological Factors

History of genetic diseases was reported in four families, two of them similar to the child’s pathology. Relatives of three subjects were diagnosed with radial polydactyly and one with arthrogryposis. Finally, 10 (37%) of the parents mentioned that at least 1 member of the household suffers of chronic conditions (i.e., diabetes mellitus, hypertension). Table 2 shows a detailed description of factors that are known to be determinants of health.

**Table 2:** **Factors determinants of health derived from interviews to 27 parents of children with congenital upper limb differences in La Mascota Hospital**.

Environmental factors	*N*	%	Economic factors	*N*	%	Individual factors		*N*	%
**Water for consumption**			Person in charge of expenses			Nutrition			
Accessible	19	70	Father	19		Balanced		13	48
Less accessible	5	19	Full-time employee	13	68	Regular		9	33
Inaccessible	3	11	Half-time/*per diem*	6	32	Not balanced		5	19

**Electricity**			Household monthly income			Weight			
Possess electricity	24	89	Stable	13	48	Normal weight		12	44
Irregular service	2	7	Regular stability	5	19	High or low weight		15	56
No electricity	1	4	Unstable	9	33	Obesity or desnutrition		0	0.0

**Housing**			Balance of monthly income			Vigorous physical activity	
Adequate materials	22	82	Positive (savings)	4	15	Adequate 3 h/week		20	74
Regular materials	3	11	Neutral	12	44	Regular 1–2 h/week		4	15
Bad housing materials	2	7	Negative (debt)	11	41	Low or none: <1–0 h/week		3	11

**Transportation**						Smoking in household		
Accessible	14	52	Social factors			Nobody smokes		22	82
Somewhat accessible	6	22	Highest level of education			Smoke outside		2	7
Not accessible	7	26	Superior studies	7	26	Smokes inside		3	11
Km Distance from La Mascota (median, IQR)	28	8–100	Primary complete secondary	19	70	Alcohol in household			

**Security**			Primary incomplete or no education	1	4	Nobody consumes alcohol		16	60
Secure neighborhood	18	67	Family time together			Socially 1 day a week		9	33
Minor crimes	7	26	Share time together	23	85	Consumption several days a week		2	7
Major crimes	2	7	Busy, little time together	3	11	Personal factors	

**Health coverage**			Very little or no time together	1	4
Private insurance covers expenses	0	0	Culture and tradition			Genetic diseases			
Public institution covers all expenses	10	37	Highly involved	8	30	No family history of genetic disease		23	86
Public partially covered expenses	15	56	Sometimes involved	10	37	Family history but not direct family		2	7
Pays out of pocket most expenses	2	7	Not involved	9	33	Family history of genetic condition		2	7

**Accessibility to basic health services**			Religion			Medical conditions in household			
Accessible and efficient	19	70	Important and practiced	16	59	Only minor conditions		14	52
Less accessible with delays	6	23	Sometimes practiced	8	30	Acute diseases (viral diseases)		3	11
Inaccessible	2	7	Not practiced	3	11	Chronic conditions		10	37

**Access to hand surgeons**							
Accessible	1	4							
Less accessible	4	15							
Not accessible	22	81							

*Managua, Nicaragua, 2016*.

### Health Needs Prioritization

Parents were asked to prioritize the following needs: access to specialized orthopedic care and hand surgeons, improvement of basic health services, family income, employment opportunities, and improvement of child’s upper extremity function. In addition to what parents considered important, the process of prioritization included the input of other stakeholders, in this case health-care providers from La Brigada and local physicians from La Mascota Hospital. They rated the needs considering the internal capacity to develop potential interventions, in such a way that needs were relevant for the community and attainable by La Brigada. Table 3 shows the variables of the prioritization based on the Hanlon method and the final ranking of needs.

**Table 3:** **Prioritization of needs according to Hanlon method**.

Most important perceived need (order in interview)	Size	% negative outcome	Seriousness	% parent priority	Effectiveness	PEARL	Priority score	Rank
Access to specialists	10	81.5	8	21.7	8	1	208	1
Access to rehabilitation	6	7.4	5	2.2	8	1	128	2
Improve UE function.	5	7.7	6	7.6	5	1	85	3
Transportation	9	25.9	6	6.5	3	1	63	4
Physical activity	7	11.1	5	3.3	3	1	51	5
Family support	5	3.7	5	1.1	3	1	45	6
Employment	9	31	6	9.8	2	1	42	7
Diseases in the family	9	37	5	2.2	2	1	38	8
Weight	0	0	5	4.4	2	1	20	9
Access to basic health care	6	7.4	7	13	2	0	0	10
Culture, traditions, and religion	9	33.9	0	0	1	0	0	10
Education	5	3.7	6	7.6	2	0	0	10
Habits: smoke, alcohol	7	11.1	5	1.1	2	0	0	10
Housing	6	7.4	6	6.5	0	0	0	10
Money expenses	9	40.7	5	1.1	0	0	0	10
Money income	9	33.3	7	10.8	0	0	0	10
Nutrition	8	22.	6	5.4	2	0	0	10
Security	6	7.4	5	1.1	0	0	0	10
Water, electricity	7	11.1	5	2.2	0	0	0	10

## Discussion

La Brigada, under the auspices of HVO, established continuity in volunteering efforts for children with hand problems in Managua, Nicaragua, in 2009. Through 16 biannual trips, La Brigada has not only provided specialized health-care services to patients with upper limb pathology but also has formed a partnership with Nicaraguan colleagues which has proven to be effective strengthening local health-care efforts of delivering surgical care to children with hand pathologies. By training a local surgeon, the partnership aims to cover a gap in the shortage of specialists in Nicaragua, addressing one of the major problems in low- and middle-income countries.

Due to this collaborative alliance, La Brigada has gained the confidence of La Mascota Hospital administration to extend efforts beyond volunteering and training, by exploring health promotion opportunities through research endeavors that improve other aspects of health. In order to promote health, however, the first step is to gain a deeper understanding of what the patient needs and to determine if health-care professionals have the capacity to make the improvements the patient and the family request. In addition, this analysis also made clear the role of social determinants of health in relationship with access to health-care services. Children with congenital upper limb differences are more susceptible to long-term disability due to physical impairments (9), but in addition, their development might be more under the influence of their socioeconomic environment (8). For that reason, the partnership aims to improve health care for children by determining the unmet health needs of children with congenital upper limb differences and the understanding of social determinants of health with the goal of developing a plan of action that successfully addresses factors within the context of the priorities of the community. Considering the opinion of all stakeholders (parents, patients, and members of la Brigada de las Manos as well as Nicaraguan colleagues), the assessment revealed the most important needs that are relevant for children with congenital differences and are considered achievable to the health-care team, which are as follows:

### *Health Need 1*: Improving Access to Orthopedic Surgeons and Hand Surgeons

Respondents uniformly expressed that the most important health need is related to their child’s condition, ranking first the need for hand surgeons who specialize in the treatment of congenital upper limb differences. Parents favorably viewed visiting surgeons from La Brigada and were very appreciative of their services; however, the priority of this need reinforces the importance of training local surgeon(s) who can serve these patients throughout the year. To that end, La Brigada has prioritized training a local orthopedic surgeon the necessary skills to perform pediatric hand surgical procedures independently. This training strategy to date has proved to be successful, as this physician has carried out hand surgeries in children with congenital pathologies such as polydactyly and syndactyly when La Brigada is not present with favorable results. Although this training will make specialized care more available at La Mascota Hospital, it does not meet the needs of communities that are distant to Managua.

### *Health Need 2*: Improving Access to Rehabilitation and Therapists

The second need derived from the analysis is that patients perceived the need to increase in the capacity of rehabilitation services, especially the ability to fabricate splints with thermoplastic materials. Since La Brigada started recurrent trips to La Mascota, the fabrication of splints has relied on donations of splint material from volunteers and requires the guidance of U.S. therapists. Through continuous skills training, a pool of local therapists has been able to gain proficiency in splint fabrication to cover the basic needs of patients in La Mascota Hospital. While this method has proven effective for training purposes, donated materials do not meet the local demand of the locally trained hand surgeon and therapists. This area of need requires more institutional compromise to extend the partnership to areas beyond training of local therapists; with institutional negotiations an agreement could be reached with industry that can provide materials at low cost. There is the need to establish a local institutional mechanism that guarantees the provision of resources instead of relying in donations.

### *Health Need 3*: Improving Upper Extremity Function

As result of the examination of PROMIS questionnaires, patients and parents acting as their proxy expressed the importance of improving child’s upper extremity function. It is not surprising that upper extremity function is affected in this patient’s population. Prior research has shown a wide spectrum of outcomes depending on the type of congenital difference and the level of involvement. For example, patients with unilateral below-the-elbow deficiency exhibit deficits in upper extremity function when compared with controls (8, 15, 16). Parents believed that improving function would optimize performance for future employment opportunities. A congenital condition is sometimes stigmatized (17), and if children are perceived as unable to perform a manual task, they are at a relative disadvantage compared with same age peers.

### *Health Need 4*: Improve Access to Transportation

Parents frequently complained of the difficulty accessing surgical services due to several factors related to distance, poor roads, lack of suitable transport, and cost related to accessing the hospital. The first inconvenience is the distance they have to travel from their communities, as on average, families traveled 57 km to get to La Mascota Hospital. Second is the cost, because public transportation is infrequent in rural areas, a round trip to the hospital is lengthy, and some families have to travel 1 day in advance and stay overnight in a hotel in order to arrive in time for their child’s appointment, which increases the cost of the trip. Parents may also have to take unpaid time off to travel with their child to the appointment.

### Health Need 5: Increase physical activity

Overall, parents expressed that their children were active; however, 25% of them are not getting the necessary physical activity recommended by WHO of 3 h of moderate to intense physical activity per week. In addition, parents expressed that patients were not involved in many sports that required upper limb involvement due to the perceived physical limitation. There were several reasons for this, but the most important were their fear of rejection by other children, and they felt unable to practice certain sports because of their physical impairment.

### Lessons Learned

After analyzing the needs of families and considering the capacities of La Brigada, the next steps this partnership between American and Nicaraguan health providers became apparent. Based on the findings, possible interventions were outlined to improve health of patients with congenital upper limb differences. La Brigada is already addressing the most important need by providing pediatric hand surgery training to a local orthopedic surgeon who will care for patients with congenital differences between la Brigada visits, increasing access to specialists. This surgeon, in turn, works with Nicaraguan orthopedic surgery residents from three local training programs. Similar “train the trainer” strategies have proved effective in orthopedic surgery. One example of the initiatives on non-surgical care has been physician education in the Ponseti method to treat clubfeet in developing countries (18). Similarly, private non-profit organizations have improved the availability and quality of health care in developing countries through the education of local health-care workers—from general surgeons to clinical officers to orthopedic assistants—in musculoskeletal conditions (19).

In a similar effort, the need for physical therapists has also been addressed as part of the ongoing efforts of La Brigada. Physical therapists in La Mascota hospital are being trained in the fabrication of splints due to the high demand of services for patients with congenital differences and neuromuscular problems. However, the need to make splint materials more available to local therapists in La Mascota persists. Local therapists currently rely on donated material, but this is not sustainable in the long term. In response to results from this needs assessment, La Brigada’s therapy director will work to engage the hospital’s administration and local institutions to develop purchase mechanisms that will allow the acquisition of a steady supply of thermoplastic materials, thus making splints more available for the community.

In response to the need for increased access to orthopedic services in remote locations, La Brigada plans to initiate an outreach clinic in Leon, a large population center 75 km from Managua, so that only patients who need surgical care (20–25% of those seen in the La Brigada clinics) will have to travel to La Mascota Hospital. Similar strategies to deliver care in rural areas have been implemented in other areas of medicine with promising results, as is the case of mobile clinics dedicated to cancer screening (20), dental care (21), and maternal and child services (22).

Finally, in an effort to improve upper extremity function, physical activity, and sports participation, La Brigada will work to develop an intervention for skills training focused in promoting function enhancement and activity performance that will lead to increased physical capacities of patients with congenital upper limb differences. Several young adults with congenital hand differences have recently joined La Brigada as volunteers; the development of skills training programs may be a possible role for them on future trips as they have shown to be successful in people with disabilities (23, 24).

## Conclusion

The purpose of this health needs assessment was to discover the most important health needs of children with congenital upper limb differences and to propose interventions to address their needs, considering the opinions of the community. An approach that takes into account social determinants of health and considers the opinion of families is important to develop effective and long-lasting interventions that suit the social and economic environment of the family. In addition, the case study highlighted the importance of having organized partnerships that have built a long-standing relationship of trust with local authorities and community members and have committed resources to the improvement of health of this vulnerable population. An assessment of health needs should be considered one of the first activities of global health partnerships, because this process guides the efficient allocation of resources and maximizes results, taking in account what the community needs. As result of this case study, we learned that the most important need for patients and families is improving access to specialized care for their child’s condition, including hand surgeons and rehabilitation services that will improve child’s upper extremity function. In addition, parents expressed difficulty accessing the hospital from distant places and they will benefit from strategies that address this problem. Finally, there was the need to improve physical activity and sport participation as means to increase social inclusion and acceptance in their community. Health-care providers from La Brigada and Nicaraguan partners in response to this assessment have identified potential solutions to these needs and are currently working in future interventions.

## Ethics Statement

This study was carried out in accordance with the recommendations of the Human Research Protection Office with written informed consent from all subjects. All subjects gave written informed consent in accordance with the Declaration of Helsinki. The protocol was approved by the University of Massachusetts—Amherst Institutional Review Board and La Mascota Hospital Director.

## Author Contributions

MC: lead author, study conception and design, compiled/collected data, data analysis, manuscript writing, critical revision of the manuscript, and final approval of the version being published. JR-R and GR-Z: co-authors, study conception, interpretation of data for the work, critical revision of the manuscript, final approval of the version being published. MJ: senior author, study conception and design, interpretation of data for the work, critical revision of the manuscript, final approval of the version being published, critical revision of the manuscript.

## Conflict of Interest Statement

The authors declare that the research was conducted in the absence of any commercial or financial relationships that could be construed as a potential conflict of interest.
